# Enhancement of methane production from 1‐hexadecene by additional electron donors

**DOI:** 10.1111/1751-7915.12886

**Published:** 2017-12-07

**Authors:** A. M. S. Paulo, A. F. Salvador, J. I. Alves, R. Castro, A. A. M. Langenhoff, A. J. M. Stams, A. J. Cavaleiro

**Affiliations:** ^1^ Centre of Biological Engineering University of Minho Braga Portugal; ^2^ Sub‐department of Environmental Technology Wageningen University & Research Wageningen The Netherlands; ^3^ Laboratory of Microbiology Wageningen University & Research Wageningen The Netherlands; ^4^ Present address: Centre of Biotechnology and Fine Chemistry Catholic University of Portugal Porto Portugal

## Abstract

1‐Hexadecene‐contaminated wastewater is produced in oil refineries and can be treated in methanogenic bioreactors, although generally at low conversion rates. In this study, a microbial culture able to degrade 1‐hexadecene was enriched, and different stimulation strategies were tested for enhancing 1‐hexadecene conversion to methane. Seven and three times faster methane production was obtained in cultures stimulated with yeast extract or lactate, respectively, while cultures amended with crotonate lost the ability to degrade 1‐hexadecene. Methane production from 1‐hexadecene was not enhanced by the addition of extra hydrogenotrophic methanogens. Bacteria closely related to *Syntrophus* and *Smithella* were detected in 1‐hexadecene‐degrading cultures, but not in the ones amended with crotonate, which suggests the involvement of these bacteria in 1‐hexadecene degradation. Genes coding for alkylsuccinate synthase alpha‐subunit were detected in cultures degrading 1‐hexadecene, indicating that hydrocarbon activation may occur by fumarate addition. These findings are novel and show that methane production from 1‐hexadecene is improved by the addition of yeast extract or lactate. These extra electron donors may be considered as a potential bioremediation strategy of oil‐contaminated sites with bioenergy generation through methane production.

## Introduction

Linear alpha olefins (LAO) are obtained from crude oil refinery and consist of unsaturated straight‐chain hydrocarbons containing a double bond at the primary or alpha position (American Chemistry Council, 2006). Global LAO consumption has increased at an average annual rate of 5.6% from 2012 to 2016 and is expected to continue at 3.7% average annual rate until 2021 (IHS Markit, 2017). 1‐Hexadecene, a LAO with 16 carbon atoms, is abundant (i.e. 60–68%, Chevron Phillips Chemical Company, 2013) in LAO commercial blends that are used in the production of tanning oils, synthetic fatty acids and drilling fluids for off‐shore oil exploration (Herman and Roberts, 2005). During 1‐hexadecene production in petrochemical plants, contaminated wastewater is generated, which can be treated by anaerobic digestion. This leads to the production of methane that is stored and used as biofuel (Scherr *et al*., 2012).

Methanogenic degradation of 1‐hexadecene is poorly described, and only two enrichment cultures degrading this compound were reported (Schink, 1985). Methanogenic archaea resembling *Methanospirillum* and *Methanosaeta* could be identified, but no information on the bacterial composition was reported. The microbiology and biochemistry of aliphatic hydrocarbons degradation under methanogenic conditions have been mainly studied with hexadecane as model compound, a saturated C_16_ straight‐chain hydrocarbon (Zengler *et al*., 1999; Jones *et al*., 2008; Siddique *et al*., 2012). Syntrophic bacteria assigned to *Syntrophaceae* were suggested to degrade hexadecane to acetate and hydrogen, which are consumed by methanogens (Gray *et al*., 2011; Embree *et al*., 2015; Jiménez *et al*., 2016; Wawrik *et al*., 2016). Although still not known, it is unlikely that these bacteria can degrade hexadecene, as the only two cultures described to degrade 1‐hexadecene were not able to use hexadecane (Schink, 1985).

Alkene and alkane conversion to methane is a very slow process – the two cultures reported by Schink (1985) took approximately 100 days to convert 0.174 mmol of 1‐hexadecene to methane, and 810 days of incubation were needed by a methanogenic enrichment for the degradation of 0.59 mmol of hexadecane (Zengler *et al*., 1999). Therefore, strategies to improve the process efficiency are needed. For example, the addition of easily degradable carbon sources such as acetate, lactose or methanol improved the bioremediation of hydrocarbon‐contaminated sediments (Dell *et al*., 2012; Zhang and Lo, 2015).

In this work, enrichment cultures with 1‐hexadecene or hexadecane were started using anaerobic sludge as inoculum, with no previous adaptation to petroleum hydrocarbons. Methane was produced from 1‐hexadecene, but not from hexadecane. To increase 1‐hexadecene conversion rates, stimulation by addition of hydrogenotrophic methanogens or co‐substrates was tested. Methane production, 1‐hexadecene degradation and microbial community composition were studied.

## Results and discussion

An enrichment culture with the ability to degrade 1‐hexadecene under methanogenic conditions was obtained from anaerobic granular sludge after three successive transfers. Methane production by culture He(3) proceeded slowly and reached 6.9 mM after approximately 10 months of incubation (Table 1), which corresponds to 96% of the stoichiometric value expected from the initial 1‐hexadecene concentration of 0.6 mM (Equation 1).

(Equation 1) $$C_{16}H_{32}+8H_{2}O\to 12{\text{CH}}_{4}+4{\text{CO}}_{2}$$

In the cultures amended with hexadecane, methane production decreased with each transfer (Table 1). After three successive transfers (culture H(3)), almost no methane was produced during 10 months of incubation. Therefore, this enrichment was not continued and further work was performed only with 1‐hexadecene‐degrading cultures.

**Table 1:** Methane production, hydrocarbon biodegradation and 1‐hexadecene concentration in the enrichment cultures

Code	Time (months)	Methane (mM)	Biodegradation (%)^a^	1‐Hexadecene (mM)
Before stimulation				
H(1)	2.0	16.7	227	n.a.
H(2)	1.2	1.2	16	n.a.
H(3)	1.6	0.1	1	n.a.
	6.9	0.1	1	n.a.
	10.0	0.0	1	n.a.
Before stimulation				
He(1)	2.0	8.6	120	n.d.
He(2)	1.2	5.4	75	n.d.
He(3)	1.6	0.4	5	n.d.
	6.9	2.4	33	n.d.
	10.0	6.9	96	n.d.
He(4)	3.0	1.2 ± 0.2	16 ± 2	n.d.
	6.8	3.7 ± 0.5	51 ± 6	n.d.
He(5)	5.5	1.5	21	n.d.
After stimulation with methanogens				
He‐Mf(4)	3.0	1.2 ± 0.2	17 ± 3	n.d.
	6.8	2.9 ± 0.3	40 ± 5	n.d.
After stimulation with yeast extract				
He‐WOY(7)	5.2	12.4 ± 0.4	103 ± 3	0.0±0.0
He‐WOY(9)	2.7	7.6 ± 0.6	64 ± 5	n.d.
He‐WOY(10)	1.9	5.5	46	n.d.
After stimulation with lactate				
He‐WOL(7)	5.5	6.0 ± 1.2	50 ± 10	0.4 ± 0.0
After stimulation with crotonate				
He‐WOC(7)	7.0	0.5 ± 0.3	4 ± 2	1.0 ± 0.1

n.d., not determined; n.a., not applicable.

a Calculated considering the methane produced, and the maximum theoretical methane production expected (i.e. 7.2 and 12 mM methane from 0.6 and 1 mM of 1‐hexadecene, respectively; 7.4 and 12.25 mM methane from 0.6 and 1 mM of hexadecane respectively (Dolfing *et al*., 2008)).

### Addition of hydrogenotrophic methanogens

The slow biodegradation of 1‐hexadecene to methane by the enriched culture He(3) was the motivation for performing the stimulation assays. Methanogenic biodegradation of alkanes is considered a hydrogen/formate‐dependent syntrophic process (Jiménez *et al*., 2016), and the presence of hydrogenotrophic methanogens in the hexadecene‐degrading enrichment cultures developed by Schink (1985) suggests that this may be also the case for 1‐hexadecene. We used *Methanobacterium formicicum* to bioaugment our assays, to assure that thermodynamics constrains caused by hydrogen accumulation would not limit the complete conversion of 1‐hexadecene to methane. However, the addition of this active hydrogenotrophic partner did not enhance methane production, as He‐Mf(4) cultures performed similarly to He(4) cultures with methane reaching 2.9 ± 0.3 and 3.7 ± 0.5 mM in 7 months respectively (Table 1). This corresponds to 40% and 51% of the theoretical expected methane production. No VFA were detected in these assays. In the blanks, where no substrate was added, methane was not produced. Addition of *M. formicicum* was not a successful stimulation strategy probably due to an inhibitory effect of 1‐hexadecene towards this methanogen, or because methanogens activity was not the limiting step in 1‐hexadecene degradation by these cultures.

### Stimulation by additional electron donors

In this study, yeast extract, lactate and crotonate were chosen as additional electron donors to stimulate the key players involved in 1‐hexadecene biodegradation. Two transfers were performed with 1‐hexadecene and the additional electron donors, after which the cultures were incubated again with 1‐hexadecene only (He‐WOY(7), He‐WOL(7) and He‐WOC(7)) (Fig. S1).

**Figure 1:**
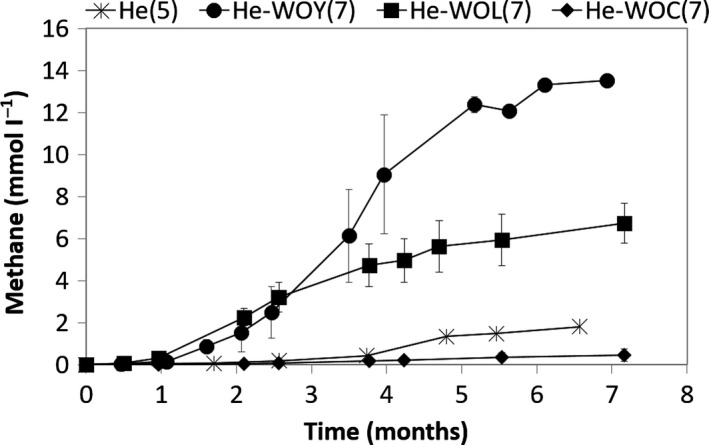
Methane production in cultures incubated only with 1‐hexadecene. He(5) – non‐stimulated enriched culture, He‐WOY(7) – enrichment culture after stimulation with yeast extract, He‐WOL(7) – enrichment culture after stimulation with lactate, He‐WOC(7) – enrichment culture after stimulation with crotonate.

Short lag phases of one month preceded methane production by the yeast extract‐ or lactate‐stimulated cultures, while more than 3 months were necessary to detect methane in the non‐stimulated culture He(5) and almost no methane could be quantified in incubations with the culture that had been previously amended with crotonate (Fig. 1). The inability of culture He‐WOC(7) to degrade the alkene was confirmed by the quantification of 1.0 ± 0.1 mM of 1‐hexadecene at the end of the assays, which corresponds to the added concentration at the beginning of the incubation (Table 1). In this work, crotonate addition intended to stimulate the growth of syntrophic bacteria, as some species can be grown in pure culture on this substrate. For example, *Syntrophus* species, *Smithella propionica* and some *Syntrophomonas* species are capable of fermenting crotonate (Beaty and McInerney, 1987; Jackson *et al*., 1999; Liu *et al*., 1999). Our results suggest that during incubation with crotonate and 1‐hexadecene (enrichment cultures He‐C(5) and He‐C(6)), the enrichment lost its ability to convert 1‐hexadecene (as only carbon source) to methane, probably due to a higher enrichment of crotonate degraders over 1‐hexadecene degraders.

After 5 months of incubation, complete 1‐hexadecene conversion to methane was achieved by He‐WOY(7), and 50% conversion by He‐WOL(7), while He(5) produced only 21% of the expected methane (Fig. 1, Table 1). This shows that the presence of yeast extract or lactate during two successive generations accelerated the methane production from 1‐hexadecene, respectively, 7 or 3 times, compared with the non‐stimulated culture. No VFA were detected during the incubations. Yeast extract is known to provide micronutrients and vitamins, and it is also a common carbon and energy source for microorganisms (Khelaifia *et al*., 2013). In this work, yeast extract was added as a non‐specific co‐substrate to increase the microbial abundance and diversity in the culture, as bioremediation strategies that promote high bacterial diversity can be more efficient than those targeting specific taxa (Dell *et al*., 2012). Lactate can be converted to acetate and hydrogen by different syntrophic bacteria, which can be further degraded to methane by hydrogenotrophic and acetoclastic methanogens (Junicke *et al*., 2015). Therefore, lactate addition aimed the stimulation of syntrophic communities.

Undoubtedly, yeast extract was the best co‐substrate for accelerating 1‐hexadecene biodegradation. This effect was even more pronounced in cultures He‐WOY(9) and He‐WOY(10) that could convert approximately 50% of the 1‐hexadecene to methane in 2 months (Table 1), which was much faster than all the other cultures described in this work. The addition of fermented yeast extract (addition of cofactors) did not increase methane production (Fig. S3). This shows that the positive effect of yeast extract was due to a faster growth of the microbial community resulting from its consumption as additional carbon source, and not due to the presence of extra cofactors.

Biodegradation of yeast extract, lactate and crotonate was verified in control assays prepared without 1‐hexadecene (Fig. S2). The theoretical methane production expected from yeast extract, lactate and crotonate was 8.7, 6.8 and 9.2 mM respectively. More than 96% of the theoretical methane production was attained after 48 days of incubation in yeast extract, and 77 days in lactate and crotonate (Fig. S2a). At the end of the incubations, lactate and crotonate were not detected, and only residual amounts of yeast extract were present in the medium (determined by soluble chemical oxygen demand (COD) measurements, Fig. S2b). No methane was produced in the blank assays prepared without any added substrate.

### Microbial community analysis

All 1‐hexadecene‐degrading cultures presented high microbial diversity. Changes in microbial abundance and composition were detected between non‐stimulated and stimulated cultures (Table 2 and Table S2). In the stimulated cultures, the growth of bacteria was promoted over methanogenic archaea, with relative percentages increasing from 48% up to 87% (Table 2). Bacterial groups *Syntrophaceae*,* Clostridia*,* Synergistales* and *Spirochaetes* became more abundant in the cultures degrading 1‐hexadecene more efficiently, i.e. He‐WOY(7) and He‐WOL(7) (Table 2).

**Table 2:** Microbial composition until the genus level of non‐stimulated and stimulated 1‐hexadecene‐degrading cultures. Variation in colour intensity reflects the relative abundance of microbial groups, from light colour, less abundant, to dark colour, more abundant

Taxonomic classification						Relative abundance (%)*							
						I	He(4)	He‐WOY(7)		He‐WOL(7)		He‐WOY(10)	
	Phylum	Class	Order	Family	Genus			*a*	*b*	*a*	*b*	*a*	*b*
*Bacteria*	Total *Bacteria*					43	48	67	87	64	75	75	80
	* Proteobacteria*	*Deltaproteobacteria*	*Desulfobacterales*	*Desulfobacteraceae*	*Desulfofaba*	0.0	0.0	0.0	0.8	0.0	0.0	0.1	0.2
	* Proteobacteria*	*Deltaproteobacteria*	*Desulfovibrionales*	*Desulfovibrionaceae*	*Desulfovibrio*	0.0	1.5	0.0	0.0	0.1	0.1	0.1	0.7
	* Proteobacteria*	*Deltaproteobacteria*	*Syntrophobacterales*	*Syntrophaceae*	*Smithella*	0.0	3.4	0.0	0.0	0.0	0.0	0.0	0.0
	* Proteobacteria*	*Deltaproteobacteria*	*Syntrophobacterales*	*Syntrophaceae*	*Syntrophus*	0.0	0.0	1.6	0.5	0.8	0.5	4.0	8.2
	* Proteobacteria*	*Deltaproteobacteria*	*Syntrophobacterales*	*Syntrophaceae*	Unclassified	0.0	0.0	1.0	1.1	4.1	0.6	0.0	0.0
	* Proteobacteria*	*Deltaproteobacteria*	*Syntrophobacterales*	*Syntrophobacteraceae*	*Syntrophobacter*	0.3	0.0	0.0	0.0	0.1	0.0	0.1	0.5
	* Proteobacteria*	*Deltaproteobacteria*	*Syntrophobacterales*	*Syntrophorhabdaceae*	*Syntrophorhabdus*	0.0	0.7	0.1	0.3	0.3	0.1	0.5	0.8
	* Proteobacteria*	*Deltaproteobacteria*	*Syntrophobacterales*	Unclassified	Unclassified	0.0	0.0	0.0	0.0	0.0	0.0	0.4	0.2
	* Proteobacteria*	*Gammaproteobacteria*	*Pseudomonadales*	*Pseudomonadaceae*	*Pseudomonas*	0.0	0.0	0.0	0.0	0.0	0.0	0.0	0.2
	* Proteobacteria*	Unclassified	Unclassified	Unclassified	Unclassified	0.2	0.0	0.0	0.1	0.1	0.1	0.6	1.2
	Other *Proteobacteria*					0.9	3.7	0.6	0.2	0.6	0.2	0.1	0.2
	* Firmicutes*	*Clostridia*	*Clostridiales*	*Syntrophomonadaceae*	*Syntrophomonas*	0.0	0.0	0.0	0.0	0.0	0.0	0.0	0.6
	* Firmicutes*	*Clostridia*	Unclassified	Unclassified	Unclassified	1.2	1.7	6.0	0.0	14.9	20.0	0.4	0.1
	Other *Firmicutes*					0.8	2.4	1.3	0.1	2.0	1.4	1.2	0.6
	* Bacteroidetes*	*Bacteroidia*	*Bacteroidales*	Unclassified	Unclassified	0.1	0.0	0.7	0.1	0.6	1.6	0.1	0.0
	* Bacteroidetes*	*Cytophagia*	*Cytophagales*	*Cytophagaceae*	*Cytophaga*	1.9	0.0	0.0	0.2	0.1	0.0	0.4	0.2
	* Bacteroidetes*	*Cytophagia*	*Cytophagales*	*Cytophagaceae*	*Hymenobacter*	0.0	0.2	0.0	0.0	0.0	0.0	0.0	0.0
	* Bacteroidetes*	*Flavobacteriia*	*Flavobacteriales*	Unclassified	Unclassified	0.0	0.6	0.0	0.0	0.0	0.0	0.0	0.0
	* Bacteroidetes*	*Flavobacteriia*	Unclassified	Unclassified	Unclassified	0.0	1.2	0.0	0.0	0.0	0.0	0.0	0.0
	Other *Bacteroidetes*					0.3	0.0	0.0	0.0	0.0	0.0	0.0	0.0
	* Chloroflexi*	*Anaerolineae*	*Anaerolineales*	Unclassified	Unclassified	5.5	0.0	1.9	0.6	2.6	1.3	1.4	2.6
	* Chloroflexi*	*Anaerolineae*	Unclassified	Unclassified	Unclassified	0.0	0.0	0.5	0.1	0.8	0.3	0.1	0.5
	* Chloroflexi*	*Dehalococcoidia*	*Dehalococcoidales*	*Dehalococcoidaceae*	*Dehalococcoides*	0.0	1.5	0.0	0.0	0.0	0.0	0.0	0.0
	* Chloroflexi*	Unclassified	Unclassified	Unclassified	Unclassified	0.6	0.0	0.2	0.3	0.4	0.2	0.3	0.3
	Other *Chloroflexi *					1.8	0.0	0.0	0.1	0.1	0.1	0.2	0.2
	* Actinobacteria*	*Actinobacteria*	*Corynebacteriales*	*Corynebacteriaceae*	*Turicella*	0.0	0.0	0.0	0.0	0.0	0.0	0.0	0.9
	* Actinobacteria*	*Actinobacteria*	*Solirubrobacterales*	*Solirubrobacteraceae*	*Solirubrobacter*	0.0	0.0	0.0	0.0	0.0	0.0	0.1	0.2
	* Actinobacteria*	*Actinobacteria*	Unclassified	Unclassified	Unclassified	0.0	3.4	0.0	0.2	0. .2	0.3	0.0	0.1
	Other *Actinobacteria*					0.0	1.0	0.0	0.0	0.0	0.0	0.0	0.0
	* Synergistetes*	*Synergistia*	*Synergistales*	*Synergistaceae*	*Synergistes*	0.0	0.0	0.0	0.5	0.0	0.0	3.1	3.9
	* Synergistetes*	*Synergistia*	*Synergistales*	*Synergistaceae*	Unclassified	0.0	0.0	0.4	1.4	1.4	0.3	1.9	6.4
	* Synergistetes*	*Synergistia*	*Synergistales*	Unclassified	Unclassified	1.8	4.8	14.4	0.8	9.0	2.7	1.2	1.3
	Other *Synergistetes*					0.4	1.7	0.0	0.0	0.1	0.0	0.0	0.1
	* Spirochaetes*	*Spirochaetia*	*Spirochaetales*	*Spirochaetaceae*	*Spirochaeta*	0.0	0.0	0.0	0.1	0.2	0.1	0.1	0.7
	* Spirochaetes*	*Spirochaetia*	*Spirochaetales*	Unclassified	Unclassified	0.1	0.1	15.9	8.1	2.9	3.1	3.0	3.1
	* Spirochaetes*	*Spirochaetia*	Unclassified	Unclassified	Unclassified	0.1	0.0	0.0	0.0	0.1	0.0	0.0	0.1
	* Spirochaetes*	Unclassified	Unclassified	Unclassified	Unclassified	0.1	3.8	0.0	42.6	0.0	0.0	20.5	3.4
	* Thermotogae*	*Thermotogae*	*Kosmotogales*	*Kosmotogaceae*	*Mesotoga*	0.0	7.2	0.3	0.6	0.7	1.7	0.1	0.1
	Other *Thermotogae*					0.0	0.0	0.0	0.0	0.0	0.0	0.0	0.0
	Other Phyla					0.3	0.4	1.0	1.4	1.3	2.1	0.6	0.5
	Unclassified					26.1	8.2	20.6	27.0	20.6	38.2	34.3	42.3
*Archaea*	Total *Archaea*					57	45	33	13	36	25	25	20
	* Euryarchaeota*	*Methanobacteria*	*Methanobacteriales*	*Methanobacteriaceae*	*Methanobacterium*	10.5	31.1	6.8	0.5	5.6	1.2	0.0	0.0
	* Euryarchaeota*	*Methanobacteria*	*Methanobacteriales*	*Methanobacteriaceae*	*Methanobrevibacter*	0.1	0.0	0.0	0.0	0.0	0.0	0.0	0.0
	* Euryarchaeota*	*Methanomicrobia*	*Methanomicrobiales*	*Methanomicrobiaceae*	*Methanoculleus*	0.8	0.0	0.0	4.8	0.0	0.0	1.2	0.9
	* Euryarchaeota*	*Methanomicrobia*	*Methanomicrobiales*	*Methanomicrobiaceae*	*Methanofollis*	0.0	0.0	17.4	0.9	10.7	5.7	5.4	7.5
	* Euryarchaeota*	*Methanomicrobia*	*Methanomicrobiales*	*Methanoregulaceae*	*Methanolinea*	0.0	3.2	4.8	3.0	7.7	13.8	2.4	0.9
	* Euryarchaeota*	*Methanomicrobia*	*Methanomicrobiales*	*Methanospirillaceae*	*Methanospirillum*	0.0	0.0	0.0	1.1	0.0	0.0	5.7	3.2
	* Euryarchaeota*	*Methanomicrobia*	*Methanosarcinales*	*Methanosaetaceae*	*Methanosaeta*	45.7	10.3	3.9	1.8	11.1	3.4	9.9	6.8
	Other *Archaea*					0.0	0.6	0.2	0.4	0.6	0.4	0.5	0.3

a.Duplicates are represented by *a* and *b*.

As expected, the presence of 1‐hexadecene shaped the bacterial community already before stimulation (enrichment He(4)), which diverged from the inoculum sludge. Specifically, the relative percentage of *Proteobacteria* (9%), *Firmicutes* (4%), *Actinobacteria* (4%), *Synergistetes* (6%), *Spirochaetes* (4%) and *Thermotogae* (7%) increased (Table 2). The methanogenic community changed as well, with *Methanobacterium* genus becoming highly abundant in this enrichment (31% abundance relative to the whole microbial community, Table 2). These microorganisms are probably consuming hydrogen or formate formed during 1‐hexadecene degradation by syntrophic bacteria. After stimulation with additional electron donors, *Methanobacterium* and other hydrogenotrophic methanogens were detected, namely *Methanoculleus*,* Methanofollis*,* Methanolinea* and *Methanospirillum*. This reinforces the role of hydrogen/formate‐consuming microorganisms during anaerobic hydrocarbon degradation (Table 2). Those genera were also previously found in enrichment cultures and in environmental samples contaminated with hydrocarbons (Embree *et al*., 2013, 2015; Jiménez *et al*., 2016). The only acetoclastic methanogens detected belong to *Methanosaeta* genus, which was present in all samples.

Due to the scarce knowledge on 1‐hexadecene‐degrading microorganisms and consequently the lack of genomic information, the taxonomic identification was not possible in a significant proportion (21–42%) of the retrieved bacterial sequences. Several microorganisms capable of syntrophic metabolism, namely *Desulfomonile*,* Desulfovibrio*,* Syntrophus*,* Smithella*,* Syntrophobacter*,* Syntrophorhabdus* and *Syntrophomonas,* could be identified (Table 2 and Table S2), suggesting the syntrophic nature of anaerobic 1‐hexadecene biodegradation. Moreover, members of the *Syntrophaceae* family were enriched, particularly species related to *Syntrophus* and *Smithella* (Table 2). These species are known as putative alkane degraders and frequently detected in hydrocarbon‐contaminated environments (Cheng *et al*., 2013; Embree *et al*., 2015; Wawrik *et al*., 2016), although their involvement in 1‐hexadecene methanogenic degradation has never been reported.

In our work, eight different operational taxonomic units (OTU) were assigned to *Syntrophaceae* sharing only 91 to 98% identity with each other, which suggests that different microorganisms belonging to this family were present in the enrichment cultures (Table S3). However, accurate taxonomic identification is difficult to obtain as the identities between the OTU sequences (291 bp) and 16S rRNA gene sequences from *Syntrophus* and *Smithella* are quite similar (e.g. OTU86 shared the same identity with both genera, Table S3). This might be due to the high similarity between the 16S rRNA gene sequences of these two genera (93% identity between *Smithella propionica* (NR_024989.1) and *Syntrophus aciditrophicus* (NR_102776.1), Table S3). Sequences assigned to these genera were not found in enrichments He‐WOC(7), the ones that could no longer degrade 1‐hexadecene (Table S4), suggesting once more that these microorganisms are important for 1‐hexadecene degradation.

The presence of alkylsuccinate synthase gene *assA* in cultures He‐WOY(7) and He‐WOL(7) was detected, with five different *assA* gene sequences retrieved. This shows the potential of these microbial communities to perform hydrocarbon activation via fumarate addition as it is reported for alkanes. Those sequences were 83 to 84% identical to a clone sequence from cold marine sediments (NCBI nucleotide accession LN868298) and 76 to 78% identical to a *Smithella* clone sequence (NCBI nucleotide accession KF824850) (Table S5). These identity percentages are low, which reflects the poor knowledge on the microbiology of anaerobic 1‐hexadecene degradation. This is the first time that *assA* genes were detected in hexadecene‐degrading methanogenic enrichments, and the involvement of *Syntrophus/Smithella*‐related species in 1‐hexadecene degradation is demonstrated.

## Conclusions

This study shows that the degradation of 1‐hexadecene by a non‐adapted methanogenic community is feasible, and can be considerably enhanced by the addition of extra electron donors. Yeast extract and lactate accelerated 1‐hexadecene degradation, increasing methane production rates up to 7 and 3 times respectively. Addition of *M. formicicum* did not improve methane production from 1‐hexadecene. Syntrophic bacteria and hydrogenotrophic methanogens were enriched in 1‐hexadecene‐degrading cultures, showing the syntrophic nature of this conversion. In cultures where *Syntrophus* and *Smithella*‐like microorganisms were detected, 1‐hexadecene degradation was observed, while in cultures where these bacteria were not detected, no degradation occurred, suggesting their involvement in 1‐hexadecene degradation. The potential for a faster bioremediation strategy and bioenergy recovery in alkene‐contaminated systems is highlighted.

## Experimental procedures

### Medium composition and cultivation

Bicarbonate‐buffered mineral salt medium was prepared as described by Stams *et al*. (1993) and dispensed (50 ml) in 120‐ml serum bottles. 1‐Hexadecene (≥ 99%, Sigma‐Aldrich) was added at final concentrations of 0.6 or 1 mM, and hexadecane (≥ 99%, Sigma‐Aldrich) was added at 0.6 mM. Bottles were sealed with Viton rubber stoppers and crimp seals, and flushed several times with a mixture of N_2_/CO_2_ (80:20% v/v; 1.7 × 10^5^ Pa) by piercing the stopper with a needle. The medium was reduced with 0.8 mM sodium sulfide (Na_2_S.9H_2_O). All inoculations and transfers were performed aseptically using sterile syringes and needles. In all the experiments, the serum bottles were incubated statically, in inverted position, at 37 °C and in the dark.

### Enrichment of methanogenic cultures

Enrichments were started by adding 5 g ww (wet weight) of anaerobic granular sludge (0.08 g g^−1^ ww volatile solids (VS) concentration) from a brewery wastewater treatment plant to the serum bottles. 1‐Hexadecene or hexadecane was added at a final concentration of 0.6 mM. Successive transfers of approximately 10% (v/v) of the cultures to fresh medium were made in duplicate after confirming microbial growth and activity based on microscopic observations and methane measurements (more than 20% of the theoretical expected). Five and three transfers were made with 1‐hexadecene or hexadecane, respectively. Enrichments were coded He(x) and H(x), where x represents the number of transfers, and He or H the hydrocarbon added, i.e. 1‐hexadecene or hexadecane, respectively.

### Addition of hydrogenotrophic methanogen

*Methanobacterium formicicum* (DSM 1535^T^) was obtained from the Deutsche Sammlung von Mikroorganismen und Zellkulturen (DSMZ; German Collection of Microorganisms and Cell Cultures, Braunschweig, Germany) and was pre‐grown as described by Sousa *et al*. (2013). Enrichment culture He(3) was transferred to four bottles containing 1‐hexadecene (0.6 mM), and two of those were bioaugmented with *M. formicicum* (10% v/v). These two enrichments series were designated He(4) and He‐Mf(4), respectively (Fig. S1). Blank assays were prepared similarly but without any added substrate. Methane production and volatile fatty acids (VFA) were measured during the incubations.

### Stimulation by additional electron donors

Stimulation of the enrichment He(4) was performed by adding yeast extract (Y, 0.5 g l^−1^), lactate (L, 4.5 mM) or crotonate (C, 4.5 mM) as additional electron donors, together with 1‐hexadecene (1 mM), during two consecutive generations (enrichment series 5 and 6, Fig. S1 and Table S1). Co‐substrate concentrations were chosen to obtain a methane production from these compounds up to a maximum of 75% of the theoretical methane production expected from the 1‐hexadecene added. These extra electron donors were added to the bottles from sterilized anoxic stock solutions. 1‐Hexadecene biodegradation to methane was evaluated in the subsequent cultures (enrichment series 7) with no addition of extra electron donors or carbon source. For that, five similar batch bottles were prepared from each stimulation strategy, containing 1‐hexadecene (1 mM) but without (WO) additional electron donors – coded He‐WOY(7), He‐WOL(7) and He‐WOC(7), where Y, L and C refer to yeast extract, lactate and crotonate respectively (Fig. S1, Table S1). Three bottles were used to take liquid/gas samples for methane and VFA measurements along the experiment. Furthermore, these bottles were used to determine the microbial community composition at the end of the incubations. The two other bottles were sacrificed at the end of the incubations for 1‐hexadecene quantification, to minimize sampling errors due to the hydrophobicity of this compound. In parallel, duplicate batch bottles were prepared without 1‐hexadecene and containing yeast extract, lactate or crotonate (control assays). Blank assays were performed without any carbon or energy source. Yeast extract, crotonate and lactate concentrations were measured at the beginning and end of the incubations. Yeast extract concentration was determined indirectly by measuring the soluble chemical oxygen demand (COD).

The enrichment culture He‐WOY(7) was further incubated with 1‐hexadecene (1 mM) and yeast extract (He‐Y(8)). This enrichment series was continued for two more generations with 1‐hexadecene as the only carbon and energy source (He‐WOY(9) and He‐WOY(10)) (Fig. S1). Methane production was monitored in series 8, 9 and 10, and microbial community composition was analysed in culture He‐WOY(10).

An additional experiment was performed to understand the stimulation effect of yeast extract. Degradation of 1‐hexadecene (1 mM) was evaluated in the presence of yeast extract (0.5 g l^−1^) or fermented yeast extract, the latter containing no carbon (verified by COD measurements) and being mainly a source of cofactors. Fermented yeast extract was prepared based on DSMZ medium 119. Complete fermentation of yeast extract (5 g l^−1^) by anaerobic granular sludge (3 g VS l^−1^) was obtained in 120‐ml batch bottles containing bicarbonate‐buffered mineral salt medium (Stams *et al*., 1993) and a mixture of N_2_/CO_2_ (80:20% v/v) in the headspace. Culture He‐WOY(7) was used as inoculum for this test (Fig. S1). Soluble COD and methane measurements were performed during the incubation to follow the complete degradation of yeast extract. The resulting supernatant (fermented yeast extract) was centrifuged, filtered (0.2 μm porosity) and added to the assays at a final concentration equivalent to 0.5 g l^−1^ of yeast extract. COD was determined and a very low value of approximately 0.8 mg l^−1^ was obtained. Blanks without substrate were also prepared, and all the assays were made in duplicate. Methane production was measured along the incubation period.

### Microbial community analysis

#### DNA extraction and 16S rRNA genes sequencing

DNA was isolated from the inoculum sludge and from enrichment cultures He(4), He‐WOY(7), He‐WOL(7), He‐WOC(7) and He‐WOY(10). Homogenized aliquots of approximately 20 ml were collected and immediately frozen at −20 °C. Total genomic DNA was extracted using the FastDNA SPIN Kit for Soil (MP Biomedicals, Solon, OH), according to the manufacturer's instructions. DNA was further purified and concentrated by ethanol precipitation. 16S rRNA gene sequences were obtained by next‐generation sequencing (Illumina MiSeq, Inc. San Diego, California). All procedures, from DNA amplification, library preparation, sequencing to bioinformatics data analysis were performed at RTL (Research and Testing Laboratory, Texas, USA). DNA amplification was made using Illumina sequencing primers and the prokaryotic universal primer pair 515f/806r, targeting the V4 region of the 16S rRNA gene (Caporaso *et al*., 2011). Paired‐end reads obtained after sequencing were merged and submitted to pre‐processing. Chimeric sequences and sequences with less than 100 bp were discarded. After sequence clustering and OTU selection, microbial taxonomic assignment was performed by submitting the sequences to global alignment against a database of high‐quality sequences derived from the NCBI nucleotide database. Detailed information on the bioinformatics analysis steps can be found at the RTL website (http://rtlgenomics.com/amplicon-bioinformatics-pipeline).

All sequencing reads were submitted to the European Nucleotide Archive (ENA) under the study accession number PRJEB22083 (http://www.ebi.ac.uk/ena/data/view/PRJEB22083).

#### Alkylsuccinate synthase alpha‐subunit (assA) gene amplification, cloning and sequencing

PCR amplification of gene fragments coding for the alpha‐subunit of alkylsuccinate synthase was performed in DNA samples from He‐WOY(7) and He‐WOL(7). The primer set 7766f/8543r and Taq DNA polymerase kit (Thermo Fisher Scientific, Waltham, MA) were used, as described elsewhere (Netzer *et al*., 2013). The size of PCR amplicons was checked in agarose gel 1% (w/v) and purified using the PCR Clean‐Up kit NucleoSpin Extract II (Macherey‐Nagel, Düren, Germany) prior ligation into the pGEM Easy Vector Systems (Promega, Madison, WI). Plasmids containing the insert sequences were cloned into *Escherichia coli* NZY5α competent cells (Nzytech Genes and Enzymes, Lisbon, Portugal) following the manufacturer's instructions. Plasmids from ampicillin‐resistant transformants were amplified with the pGEM‐T vector‐targeted sequencing primers SP6 and T7. Sequencing reactions were carried out at Macrogen Europe (Amsterdam, the Netherlands). Vector and primer sequences were removed from partial forward and reverse sequences, which were further assembled using the Contig Assembly Program application included in the bioedit, version 7.0.9 software package (Huang, 1992; Hall, 1999). Sequences were run against the NCBI Nucleotide Sequence Database using BLAST (Altschul *et al*., 1990) (http://www.ncbi.nlm.nih.gov/blast/). The obtained sequences were submitted to the ENA under accession numbers ERZ477561 and ERZ477854 (study accession number PRJEB22123).

### Analytical methods

Methane concentration in the bottles’ headspace was determined by gas chromatography (Chrompack 9000) equipped with a flame ionization detector (FID) and a Carbowax 20M (80‐120 mesh, 2 m × 2 mm) column. Nitrogen was used as carrier gas at 30 ml min^−1^. Injection port, column and detector temperatures were 110 °C, 35 °C and 220 °C respectively. Liquid samples were centrifuged, filtered (0.2‐μm filter) and stored at −20 °C. Crotonate, lactate and VFA were quantified by high‐performance liquid chromatography (HPLC Jasco equipment) using a Varian MetaCarbTM 67H column (30 × 6.5 mm). The mobile phase was 5 mM H_2_SO_4_ at a flow rate of 0.6 ml min^−1^, and the column temperature was set to 60 °C with an UV detection at 210 nm. Yeast extract concentration was indirectly quantified by measuring the soluble chemical oxygen demand (COD), which was determined spectrophotometrically using COD cuvette test kits (Hach‐Lange GmbH, Dusseldorf, Germany) and a DR 2800 spectrophotometer (Hach‐Lange GmbH, Dusseldorf, Germany). For 1‐hexadecene analysis, the whole content of the batch assays was acidified at pH 2.0 with HCl and preserved at 4 °C. Each sample was sequentially extracted three times with hexane using separatory funnels, according to the procedure described by U.S. Environmental Protection Agency (USEPA, 1996). Tetradecane (C14) was used as surrogate to evaluate 1‐hexadecene extraction efficiency. The extracts were cleaned using Sep‐Pak Florisil^®^ cartridges (Waters, Milford, MA) and evaporated in TurboVap^®^ LV (Biotage, Uppsala, Sweden). 1‐Hexadecene concentration in the extracts was determined using a gas chromatograph with a flame ionization detector (GC‐MS Varian^®^ 4000), equipped with a VF‐1 ms column (30 m × 0.025 mm). Helium was used as carrier gas at a flow rate of 1 ml min^−1^. The column's temperature was kept at 60 °C for 1 min and further increased to 290 °C at 8 °C min^−1^. Detector and injector temperatures were 300 and 285 °C respectively. Undecane (C11) was used as internal standard.

## Conflict of interest

The authors declare no conflict of interest.

## Supporting information


**Fig. S1.** Scheme of the experimental procedure set‐up.
**Fig. S2.** Methane production from yeast extract, lactate or crotonate in the control assays (a) and yeast extract consumption measured indirectly by soluble COD quantification (b).
**Fig. S3.** Methane production in assays with hexadecene and yeast extract (He + Y) or hexadecene and fermented yeast extract .(He +Y_ferm_).
**Table S1.** Presence or absence of additional electron donors in enrichment cultures incubated with 1‐hexadecene.
**Table S2.** Microbial composition until the species level of non‐stimulated and stimulated 1‐hexadecene‐degrading cultures.
**Table S3.** Identity between the OTU assigned to *Syntrophaceae* family in 1‐hexadecene degrading enrichment cultures and the most related *Syntrophus* and *Smithella* 16S rRNA gene sequences.
**Table S4.** Microbial composition until the species level in the enrichment culture He‐WOC(7), after stimulation with crotonate.
**Table S5.** Identity between the *assA* gene sequences obtained from the enrichment cultures He‐WOY(7) and He‐WOL(7) and those from the NCBI nucleotide database.

## References

[mbt212886-bib-0001] Altschul, S.F. , Gish, W. , Miller, W. , Myers, E.W. , and Lipman, D.J. (1990) Basic local alignment search tool. J Mol Biol 2015: 403–410. 10.1016/S0022-2836(05)80360-2 2231712

[mbt212886-bib-0002] American Chemistry Council (2006) A comparison of the environmental performance of olefin and paraffin synthetic base fluids (SBF). URL https://www.americanchemistry.com/ProductsTechnology/Higher-Olefins/environmental-properties-of-olefin-and-paraffin-synthetic-base-fluids-SBF.pdf. [Accessed 15 September 2017].

[mbt212886-bib-0003] Beaty, P.S. , and McInerney, M.J. (1987) Growth of *Syntrophomonas wolfei* in pure culture on crotonate. Arch Microbiol 147: 389–393.

[mbt212886-bib-0004] Caporaso, J.G. , Lauber, C.L. , Walters, W.A. , Berg‐Lyons, D. , Lozupone, C.A. , Turnbaugh, P.J. , *et al* (2011) Global patterns of 16S rRNA diversity at a depth of millions of sequences per sample. Proc Natl Acad Sci USA 108: 4516–4522. 20534432 10.1073/pnas.1000080107 PMC3063599

[mbt212886-bib-0005] Cheng, L. , Ding, C. , Li, Q. , He, Q. , Dai, L.‐R. , and Zhang, H. (2013) DNA‐SIP reveals that *Syntrophaceae* play an important role in methanogenic hexadecane degradation. PLoS ONE 8: 1–11. 10.1371/journal.pone.0066784 PMC3698093 23840866

[mbt212886-bib-0006] Chevron Phillips Chemical Company (2013) AlphaPlus C16‐18 ISA 65:35. URL http://www.cpchem.com/bl/nao/en-us/specificationlibrary/AlphaPlusC16-18ISA-SalesSpec.pdf. [Accessed 15 September 2017].

[mbt212886-bib-0007] Dell, A. , Beolchini, F. , Rocchetti, L. , Marco, G. , and Danovaro, R. (2012) High bacterial biodiversity increases degradation performance of hydrocarbons during bioremediation of contaminated harbor marine sediments. Environ Pollut 167: 85–92. 22542785 10.1016/j.envpol.2012.03.043

[mbt212886-bib-0008] Dolfing, J. , Larter, S.R. , and Head, I.M. (2008) Thermodynamic constraints on methanogenic crude oil biodegradation. ISME J 2: 442–452. 18079730 10.1038/ismej.2007.111

[mbt212886-bib-0009] Embree, M. , Nagarajan, H. , Movahedi, N. , Chitsaz, H. , and Zengler, K. (2013) Single‐cell genome and metatranscriptome sequencing reveal metabolic interactions of an alkane‐degrading methanogenic community. ISME J 8: 757–767. 24152715 10.1038/ismej.2013.187 PMC3960532

[mbt212886-bib-0010] Embree, M. , Liu, J.K. , Al‐Bassam, M.M. , and Zengler, K. (2015) Networks of energetic and metabolic interactions define dynamics in microbial communities. Proc Natl Acad Sci USA 112: 15450–15455. 26621749 10.1073/pnas.1506034112 PMC4687543

[mbt212886-bib-0011] Gray, N.D. , Sherry, A. , Grant, R.J. , Rowan, A.K. , Hubert, C.R.J. , Callbeck, C.M. , *et al* (2011) The quantitative significance of *Syntrophaceae* and syntrophic partnerships in methanogenic degradation of crude oil alkanes. Environ Microbiol 13: 2957–2975. 21914097 10.1111/j.1462-2920.2011.02570.x PMC3258425

[mbt212886-bib-0012] Hall, T. (1999) BioEdit: a user‐friendly biological sequence alignment editor and analysis program for Windows 95/98/NT. Nucleic Acids Symp Ser 41: 95–98.

[mbt212886-bib-0013] Herman, D. , and Roberts, D.J. (2005) A marine anaerobic biodegradation test applied to the biodegradation of synthetic drilling mud base fluids. Soil Sediment Contam 14: 433–447.

[mbt212886-bib-0014] Huang, X. (1992) A contig assembly program based on sensitive detection of fragment overlaps. Genomics 14: 18–25. 1427824 10.1016/s0888-7543(05)80277-0

[mbt212886-bib-0015] IHS Markit (2017) Linear alpha‐olefins. URL https://www.ihs.com/products/linear-alpha-olefins-chemical-economics-handbook.html. [Accessed 15 September 2017].

[mbt212886-bib-0016] Jackson, B.E. , Bhupathiraju, V.K. , Tanner, R.S. , Woese, C.R. , and McInerney, M.J. (1999) *Syntrophus aciditrophicus* sp. nov., a new anaerobic bacterium that degrades fatty acids and benzoate in syntrophic association with hydrogen‐using microorganisms. Arch Microbiol 171: 107–114. 9914307 10.1007/s002030050685

[mbt212886-bib-0017] Jiménez, N. , Richnow, H.H. , Vogt, C. , Treude, T. , and Kruger, M. (2016) Methanogenic hydrocarbon degradation: evidence from field and laboratory studies. J Mol Microbiol Biotechnol 26: 227–242. 26959375 10.1159/000441679

[mbt212886-bib-0018] Jones, D.M. , Head, I.M. , Gray, N.D. , Adams, J.J. , Rowan, A.K. , Aitken, C.M. , *et al* (2008) Crude‐oil biodegradation via methanogenesis in subsurface petroleum reservoirs. Nature 451: 176–180. 18075503 10.1038/nature06484

[mbt212886-bib-0019] Junicke, H. , Feldman, H. , van Loosdrecht, M.C.M. , and Kleerebezem, R. (2015) Impact of the hydrogen partial pressure on lactate degradation in a coculture of *Desulfovibrio* sp. G11 and *Methanobrevibacter arboriphilus* DH1. Appl Microbiol Biotechnol 99: 3599–3608. 25472436 10.1007/s00253-014-6241-2

[mbt212886-bib-0020] Khelaifia, S. , Raoult, D. , and Drancourt, M. (2013) A versatile medium for cultivating methanogenic archaea. PLoS ONE 8: e61563. 23613876 10.1371/journal.pone.0061563 PMC3629087

[mbt212886-bib-0021] Liu, Y. , Balkwill, D.L. , Henry, C.A. , Drake, G.R. , and Boone, D.R. (1999) Characterization of the anaerobic propionate‐degrading syntrophs *Smithella propionica* gen. nov., sp. nov. and *Syntrophobacter wolinii* . Int J Syst Bacteriol 49: 545–556. 10319475 10.1099/00207713-49-2-545

[mbt212886-bib-0022] Netzer, F.Von. , Pilloni, G. , Kleindienst, S. , Krüger, M. , Knittel, K. , and Gründger, F. (2013) Enhanced gene detection assays for fumarate‐adding enzymes allow uncovering of anaerobic hydrocarbon degraders in terrestrial and marine systems. Appl Environ Microbiol 79: 543–552. 23124238 10.1128/AEM.02362-12 PMC3553772

[mbt212886-bib-0023] Scherr, K.E. , Lundaa, T. , Klose, V. , Bochmann, G. , and Loibner, A.P. (2012) Changes in bacterial communities from anaerobic digesters during petroleum hydrocarbon degradation. J Biotechnol 157: 564–572. 21939698 10.1016/j.jbiotec.2011.09.003

[mbt212886-bib-0024] Schink, B. (1985) Degradation of unsaturated hydrocarbons by methanogenic enrichment cultures. FEMS Microbiol Ecol 31: 69–77.

[mbt212886-bib-0026] Siddique, T. , Penner, T. , Klassen, J. , Nesbo, C. , and Foght, J.M. (2012) Microbial communities involved in methane production from hydrocarbons in oil sands tailings. Environ Sci Technol 46: 9802–9810. 22894132 10.1021/es302202c

[mbt212886-bib-0027] Sousa, D.Z. , Salvador, A.F. , Ramos, J. , Guedes, A.P. , Barbosa, S. , Stams, A.J.M. , *et al* (2013) Activity and viability of methanogens in anaerobic digestion of unsaturated and saturated long‐chain fatty acids. Appl Environ Microbiol 79: 4239–4245. 23645196 10.1128/AEM.00035-13 PMC3697517

[mbt212886-bib-0028] Stams, A.J.M. , Van Dijk, J.B. , Dijkema, C. , and Plugge, C.M. (1993) Growth of syntrophic propionate‐oxidizing bacteria with fumarate in the absence of methanogenic bacteria. Appl Environ Microbiol 59: 1114–1119. 16348912 10.1128/aem.59.4.1114-1119.1993 PMC202247

[mbt212886-bib-0029] U.S. Environmental Protection Agency (1996) Method 3510C: Separatory funnel liquid‐liquid extraction. Washington, DC. URL https://www.epa.gov/sites/production/files/2015-12/documents/3510c.pdf. [Accessed 15 September 2017]

[mbt212886-bib-0030] Wawrik, B. , Marks, C.R. , Davidova, I.A. , Mcinerney, M.J. , Pruitt, S. , Duncan, K.E. , *et al* (2016) Methanogenic paraffin degradation proceeds via alkane addition to fumarate by “*Smithella”* spp. mediated by a syntrophic coupling with hydrogenotrophic methanogens. Environ Microbiol 18: 2604–2619. 27198766 10.1111/1462-2920.13374

[mbt212886-bib-0031] Zengler, K. , Richnow, H.H. , Rosselló‐Mora, R. , Michaelis, W. , and Widdel, F. (1999) Methane formation from long‐chain alkanes by anaerobic microorganisms. Nature 401: 266–269. 10499582 10.1038/45777

[mbt212886-bib-0032] Zhang, Z. , and Lo, I.M.C. (2015) Biostimulation of petroleum‐hydrocarbon‐contaminated marine sediment with co‐substrate: involved metabolic process and microbial community. Appl Microbiol Biotechnol 99: 5683–5696. 25661814 10.1007/s00253-015-6420-9

