# Supplementary material for: Enhancement of methane production from 1‐hexadecene by additional electron donors
Source: Microb Biotechnol. 2017 Dec 7;11(4):657–66. doi: 10.1111/1751-7915.12886 (PMC6011941; doi:10.1111/1751-7915.12886)
Supplement: Supplementary file 1 — Fig. S1. Scheme of the experimental procedure set‐up. Fig. S2. Methane production from yeast extract, lactate or crotonate in the control assays (a) and yeast extract consumption measured indirectly by soluble COD quantification (b). Fig. S3. Methane production in assays with hexadecene and yeast extract (He + Y) or hexadecene and fermented yeast extract .(He +Yferm). Table S1. Presence or absence of additional electron donors in enrichment cultures incubated with 1‐hexadecene. Table S2. Microbial composition until the species level of non‐stimulated and stimulated 1‐hexadecene‐degrading cultures. Table S3. Identity between the OTU assigned to Syntrophaceae family in 1‐hexadecene degrading enrichment cultures and the most related Syntrophus and Smithella 16S rRNA gene sequences. Table S4. Microbial composition until the species level in the enrichment culture He‐WOC(7), after stimulation with crotonate. Table S5. Identity between the assA gene sequences obtained from the enrichment cultures He‐WOY(7) and He‐WOL(7) and those from the NCBI nucleotide database. [file MBT2-11-657-s001.docx]

**SUPPLEMENTARY INFORMATION**

**Enhancement of methane production from 1-hexadecene by additional electron donors**

Paulo AMS^1¥#^, Salvador AF^1#^, Alves JI^1^, Castro R^1^, Langenhoff AAM^1,2^, Stams AJM^1,3^, Cavaleiro AJ^1*^

^1^ Centre of Biological Engineering, University of Minho, Braga, Portugal

^2^ Sub-department of Environmental Technology, Wageningen University & Research, Wageningen, The Netherlands

^3^ Laboratory of Microbiology, Wageningen University & Research, Wageningen, The Netherlands

* Corresponding Author:

Ana J. Cavaleiro

Address: Departamento de Engenharia Biológica, Universidade do Minho, Campus de Gualtar, 4710-057 Braga, Portugal

Email: acavaleiro@deb.uminho.pt

Telephone: 00351253604423

Fax: 00351253604429


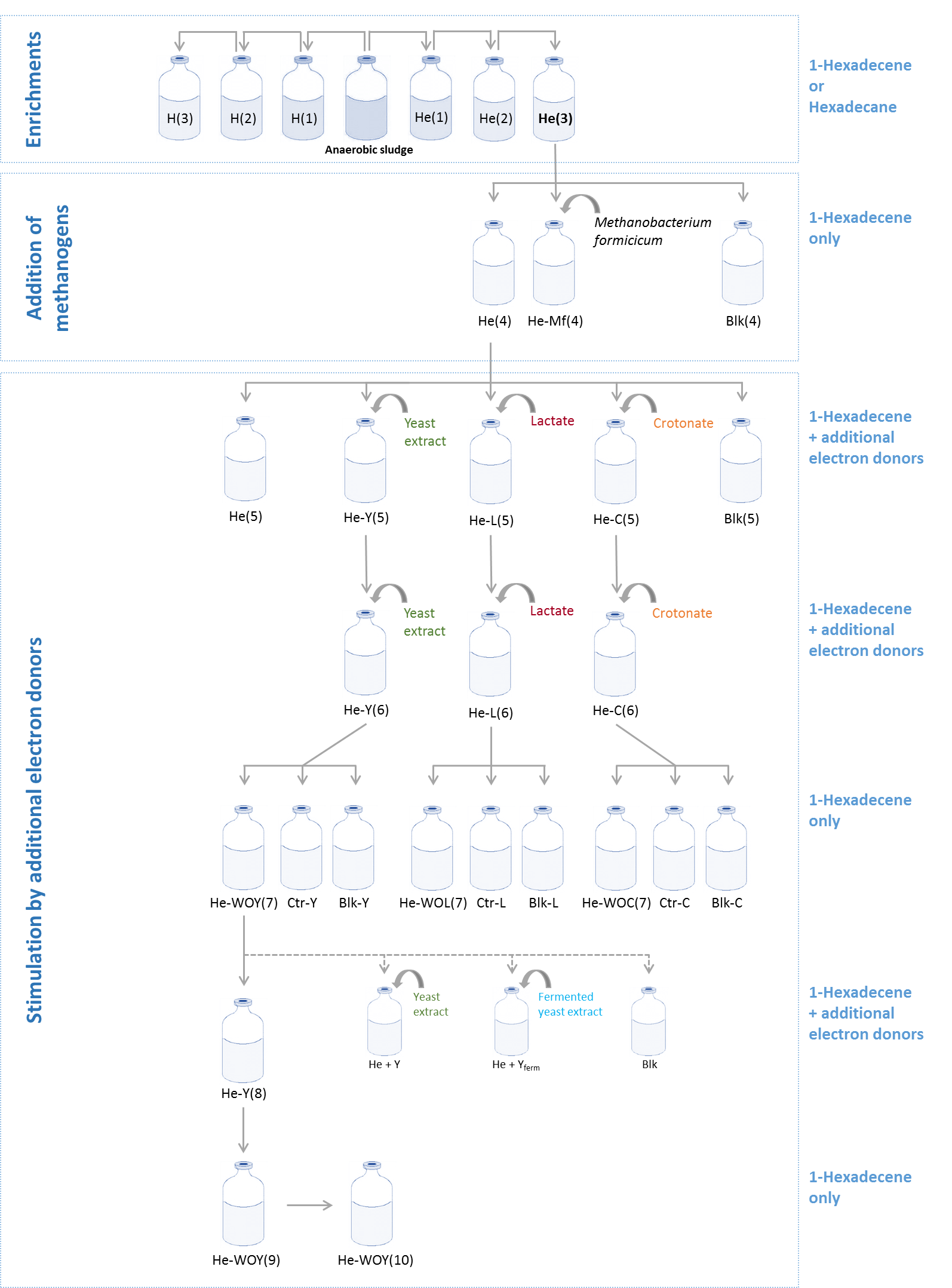


**Fig. S1** Scheme of the experimental procedure set-up. He(x) and H(x) – enrichment series with 1-hexadecene and hexadecane, respectively, where x represents the number of transfers; Mf – *Methanobacterium* *formicicum*; Y – yeast extract; L – lactate; C – crotonate; Y_ferm_ – fermented yeast extract; Blk – blank assays (without any added substrate); Ctr – control assays (amended only with the co-substrate).


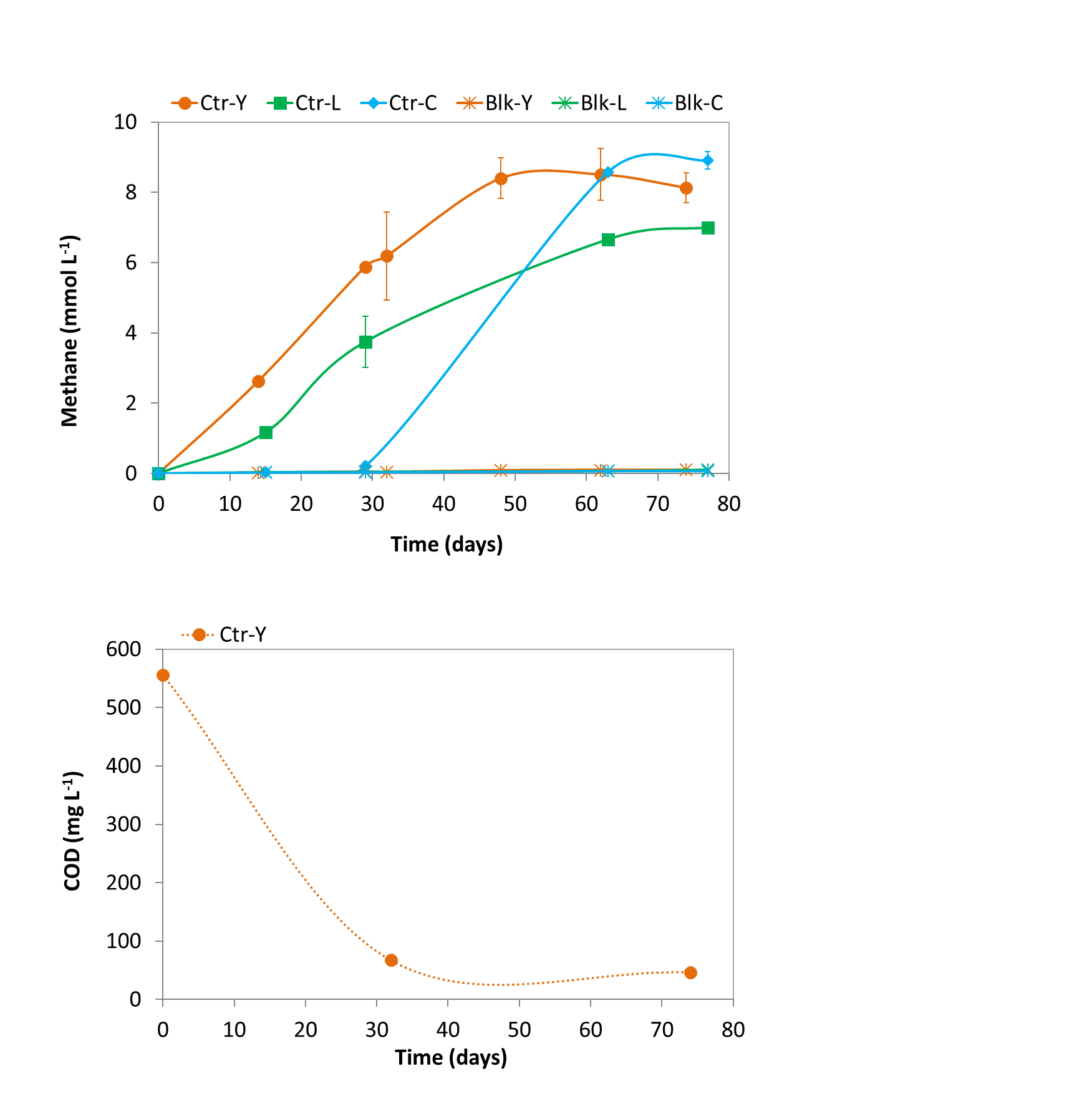


**(b)**

**(a)**

**Fig. S2** Methane production from yeast extract, lactate or crotonate in the control assays (a) and yeast extract consumption measured indirectly by soluble COD quantification (b). Ctr – control assays; Blk – blank assays; Y – yeast extract; L – lactate; C – crotonate.


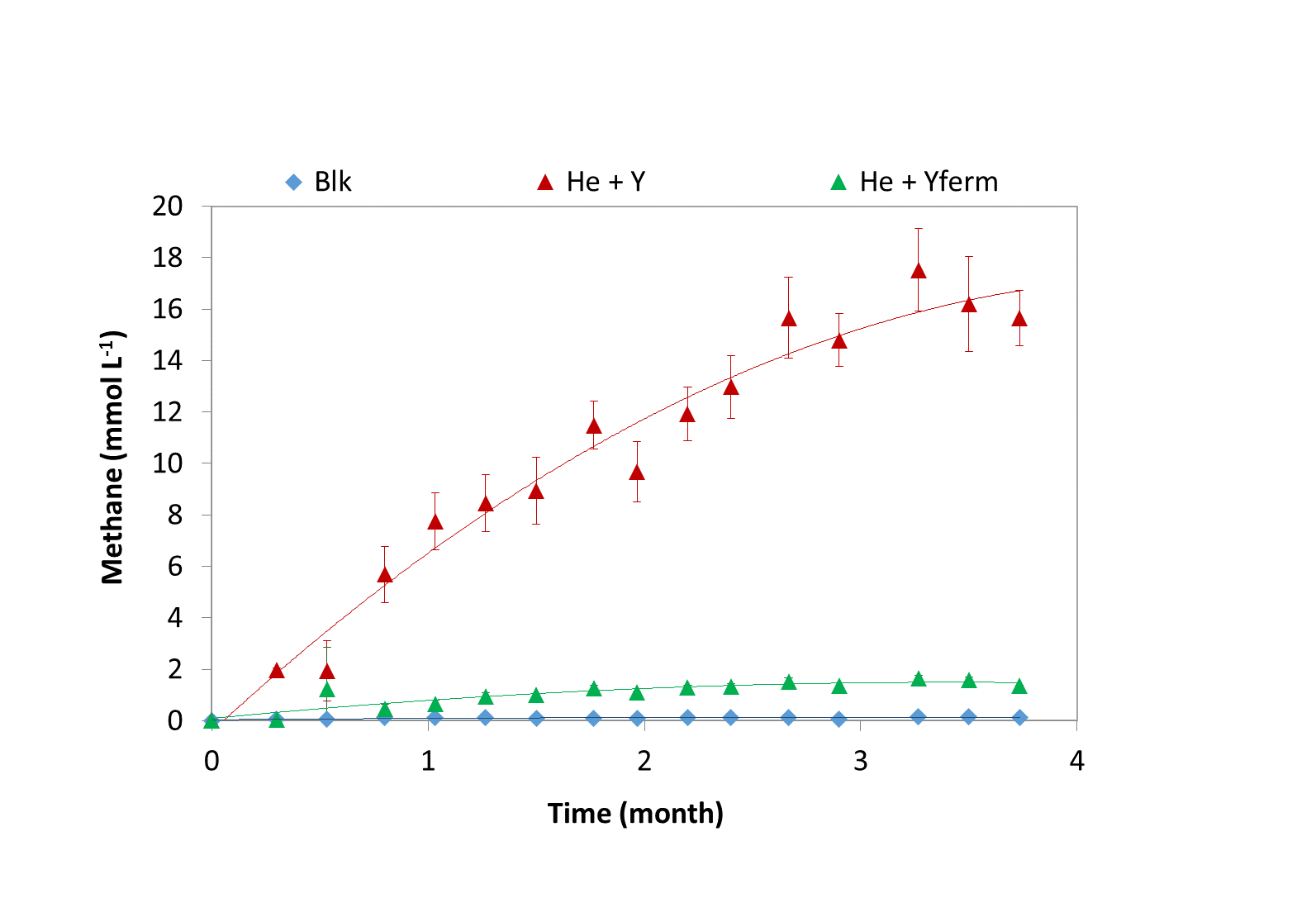


**Fig. S3** Methane production in assays with hexadecene and yeast extract (He + Y) or hexadecene and fermented yeast extract (He + Y_ferm_). Blk – blank assays.

**Table S1** Presence or absence of additional electron donors in enrichment cultures incubated with 1-hexadecene

| **Code** | **Additional electron donor** |
| --- | --- |
| He(1) | --- |
| He(2) | --- |
| He(3) | --- |
| He(4) | --- |
| He(5) | --- |
| He-Y(5) | Yeast extract |
| He-L(5) | Lactate |
| He-C(5) | Crotonate |
| He-Y(6) | Yeast extract |
| He-L(6) | Lactate |
| He-C(6) | Crotonate |
| He-WOY(7) | --- |
| He-WOL(7) | --- |
| He-WOC(7) | --- |
| He-Y(8) | Yeast extract |
| He-WOY(9) | --- |
| He-WOY(10) | --- |

**Table S2** Microbial composition until the species level of non-stimulated and stimulated 1-hexadecene-degrading cultures. Variation in color intensity reflects the relative abundance of microbial groups, from light color, less abundant, to dark color, more abundant **[EXCEL FILE]**

**Table S3** Identity between the OTU assigned to *Syntrophaceae* family in 1-hexadecene degrading enrichment cultures and the most related *Syntrophus* and *Smithella* 16S rRNA gene sequences

|  | OTU assigned to *Syntrophaceae* family in 1-hexadecene degrading enrichment cultures | | | | | | | | Most similar 16S rRNA sequences from culturable microorganisms | | |
| --- | --- | --- | --- | --- | --- | --- | --- | --- | --- | --- | --- |
| Identity (%) | OTU86 | OTU2536 | OTU626 | OTU1114 | OTU212 | OTU1195 | OTU31 | OTU279 | *Smithella propionica* | *Syntrophus aciditrophicus* | *Syntrophus gentianae* |
| OTU 86 | **100** |  |  |  |  |  |  |  |  |  |  |
| OTU 2536 | **97** | **100** |  |  |  |  |  |  |  |  |  |
| OTU 626 | **96** | **95** | **100** |  |  |  |  |  |  |  |  |
| OTU 1114 | **95** | **95** | **95** | **100** |  |  |  |  |  |  |  |
| OTU 212 | **94** | **93** | **93** | **92** | **100** |  |  |  |  |  |  |
| OTU 1195 | **93** | **92** | **94** | **92** | **92** | **100** |  |  |  |  |  |
| OTU 31 | **93** | **91** | **92** | **92** | **92** | **92** | **100** |  |  |  |  |
| OTU 279 | **93** | **92** | **94** | **93** | **92** | **98** | **92** | **100** |  |  |  |
| *Smithella propionica* | **96** | **95** | **97** | **96** | **93** | **93** | **93** | **93** | **100** |  |  |
| *Syntrophus aciditrophicus* | **96** | **94** | **95** | **94** | **94** | **91** | **92** | **92** | **93** | **100** |  |
| *Syntrophus gentianae* | **95** | **93** | **94** | **93** | **94** | **91** | **92** | **92** | **93** | **98** | **100** |

The percentage of identity was obtained by aligning locally the given OTU sequences and the 16S rRNA sequences of *Smithella* and *Syntrophus*. The 16S rRNA gene sequences from *Smithella propionica* (NR_024989.1), *Syntrophus aciditrophicus* (NR_102776.1) and *Syntrophus gentianae* (JQ346737.1) were chosen for comparison because, among the sequences of culturable microorganisms, they are the ones sharing the highest identities with the OTU assigned to *Syntrophaceae* that were obtained in this study.

**Table S4**

Microbial composition until the species level in the enrichment culture He-WOC(7), after stimulation with crotonate **[EXCEL FILE]**

**Table S5** Identity between the *assA* gene sequences obtained from the enrichment cultures He-WOY(7) and He-WOL(7) and those from the NCBI nucleotide database

|  | *assA* gene sequences obtained from 1-hexadecene degrading enrichment cultures | | | | | Most similar sequences in the NCBI nucleotide database | | | |
| --- | --- | --- | --- | --- | --- | --- | --- | --- | --- |
| Identity (%) | contig HeM1 | contig HeM2 | contig HeM3 | contig HeM4 | contig HeM5 | LN868298.1 | LT546452.1 | KF824850.1 | KT459343.1 |
| contig HeM1 | **100** |  |  |  |  |  |  |  |  |
| contig HeM2 | **96** | **100** |  |  |  |  |  |  |  |
| contig HeM3 | **98** | **97** | **100** |  |  |  |  |  |  |
| contig HeM4 | **97** | **96** | **99** | **100** |  |  |  |  |  |
| contig HeM5 | **98** | **96** | **99** | **98** | **100** |  |  |  |  |
| LN868298.1 | **83** | **83** | **85** | **84** | NSSF | **100** |  |  |  |
| LT546452.1 | **78** | **77** | **78** | NSSF | NSSF | NSSF | **100** |  |  |
| KF824850.1 | **78** | **76** | **78** | NSSF | NSSF | NSSF | **76** | **100** |  |
| KT459343.1 | **75** | **74** | **75** | NSSF | **75** | NSSF | **73** | **79** | **100** |

LN868298.1 Uncultured bacterium partial masD gene for methyl-alkyl succinate synthase, clone LF2_041_OTU20

LT546452.1 Uncultured bacterium partial masD gene for 1-methylalkyl succinate synthase, clone OTU_12

KF824850.1 *Smithella* sp. enrichment culture clone SCADC alkylsuccinate synthase alpha subunit (assA) gene, complete cds

KT459343.1 Prokaryote enrichment culture clone SDB_assA_OTU_5 alkylsuccinate synthase catalytic subunit (assA) gene, complete cds

NSSF: No significant similarity found
